# H^+^ and Pi Byproducts of Glycosylation Affect Ca^2+^ Homeostasis and Are Retrieved from the Golgi Complex by Homologs of TMEM165 and XPR1

**DOI:** 10.1534/g3.117.300339

**Published:** 2017-10-17

**Authors:** Nathan A. Snyder, Christopher P. Stefan, Camille T. Soroudi, Adam Kim, Carlos Evangelista, Kyle W. Cunningham

**Affiliations:** Department of Biology, Johns Hopkins University, Baltimore, Maryland 21218

**Keywords:** Calcium, homeostasis, phosphate, glycosylation, Golgi complex

## Abstract

Glycosylation reactions in the Golgi complex and the endoplasmic reticulum utilize nucleotide sugars as donors and produce inorganic phosphate (Pi) and acid (H^+^) as byproducts. Here we show that homologs of mammalian XPR1 and TMEM165 (termed Erd1 and Gdt1) recycle luminal Pi and exchange luminal H^+^ for cytoplasmic Ca^2+^, respectively, thereby promoting growth of yeast cells in low Pi and low Ca^2+^ environments. As expected for reversible H^+^/Ca^2+^ exchangers, Gdt1 also promoted growth in high Ca^2+^ environments when the Golgi-localized V-ATPase was operational but had the opposite effect when the V-ATPase was eliminated. Gdt1 activities were negatively regulated by calcineurin signaling and by Erd1, which recycled the Pi byproduct of glycosylation reactions and prevented the loss of this nutrient to the environment via exocytosis. Thus, Erd1 transports Pi in the opposite direction from XPR1 and other EXS family proteins and facilitates byproduct removal from the Golgi complex together with Gdt1.

Secretory proteins, lipids, and carbohydrates can undergo one or more cycles of glycosylation in the lumens of the Golgi complex and the endoplasmic reticulum (ER), often resulting in elaborate glycan chains (Stanley 2011). The glycosyltransferases responsible for these reactions consume nucleotide sugars such as GDP-mannose and UDP-glucose and generate nucleoside diphosphates which are rapidly converted by luminal nucleoside triphosphate diphosphohydrolases (NTPDases, or apyrases) to GMP and UMP plus inorganic phosphate (Pi) and acid (H^+^) as byproducts (Knowles 2011). Nucleotide sugar transporters embedded in the membranes of these organelles then exchange one luminal nucleoside monophosphate for one cytoplasmic nucleotide sugar to allow additional rounds of glycosylation to occur (Hirschberg *et al.* 1998). However, the fates of Pi and H^+^ byproducts of glycosylation reactions are not fully understood, and it is possible that their buildup in secretory organelles could adversely affect glycosylation reactions, sorting and trafficking of secretory proteins, and cell physiology.

Glycosyltransferases usually depend on Ca^2+^ or Mn^2+^ ions for maximal activity (Dürr *et al.* 1998). The SPCA family of P-type ATPases, which is mutated in Hailey–Hailey disease in humans (Hu *et al.* 2000; Sudbrak *et al.* 2000), directly transports Ca^2+^ and Mn^2+^ ions from the cytoplasm into the lumen of the Golgi complex to satisfy the needs of most glycosyltransferases, as well as the kexin family of proprotein convertases. The first SPCA-family Ca^2+^/Mn^2+^ pump, termed Pmr1, was discovered in budding yeast (Rudolph *et al.* 1989), and the *pmr1∆* knockout mutants exhibited strong defects in glycosylation and processing of secretory proteins in the Golgi complex, inability to proliferate in low Ca^2+^ environments, and hypersensitivity to high Mn^2+^ in the culture media (Antebi and Fink 1992; Sorin *et al.* 1997; Dürr *et al.* 1998). The *pmr1∆* mutants also exhibited mild activation of the unfolded protein response, likely because yeast naturally lacks a SERCA-family Ca^2+^ pump that supplies the ER of most other species with Ca^2+^ necessary for folding of secretory proteins (Dürr *et al.* 1998; Strayle *et al.* 1999). The deficiency of Ca^2+^ in the Golgi and ER of yeast leads to activation of the cell wall integrity MAP kinase Slt2/Mpk1, which induces expression of the K^+^ transporter Kch1 that depolarizes the cell membrane and activates a voltage-gated Ca^2+^ channel (also called HACS, and composed of Cch1, Mid1, and Ecm7) to promote Ca^2+^ influx, elevation of cytosolic free Ca^2+^ concentrations, and replenishment of the secretory Ca^2+^ pools (Locke *et al.* 2000; Bonilla *et al.* 2002; Bonilla and Cunningham 2003; Martin *et al.* 2011; Stefan and Cunningham 2013; Stefan *et al.* 2013). This mechanism of ER and Golgi Ca^2+^ homeostasis in yeast is analogous to, but mechanistically distinct from, store-operated Ca^2+^ entry mechanisms in animals (Smyth *et al.* 2010). Elevated cytosolic free Ca^2+^ in yeast also activates calmodulin and the serine/threonine protein phosphatase calcineurin, which induces expression of Pmr1 via activation of the Crz1 transcription factor (Matheos *et al.* 1997; Stathopoulos and Cyert 1997). Crz1 also induces a PMCA-family Ca^2+^ pump, termed Pmc1 (Cunningham and Fink 1994), which localizes to the limiting membrane of lysosome-like vacuoles and partially mislocalizes to the Golgi complex and ER in mutants that lack Pmr1(Marchi *et al.* 1999). Because the vacuole plays a major part in Ca^2+^ detoxification in yeast, mutants that lack Pmc1 or Crz1 exhibit strong growth defects in medium supplemented with high Ca^2+^ (Cunningham and Fink 1994; Matheos *et al.* 1997).

The yeast vacuole, like lysosomes in animals, is strongly acidified by the action of the V-ATPase, which directly transports H^+^ from the cytoplasm to the lumen (Kane 2016) The CAX-family H^+^/Ca^2+^ exchangers in the vacuolar limiting membrane utilize this pH gradient to power transport of Ca^2+^ from the cytoplasm to the vacuole lumen (Forster and Kane 2000). The first CAX-family H^+^/Ca^2+^ exchanger, termed Vcx1, was discovered in yeast based on its ability to confer Ca^2+^ or Mn^2+^ resistance when overexpressed (Cunningham and Fink 1996; Pozos *et al.* 1996). But, surprisingly, *vcx1∆* mutants exhibited little hypersensitivity to Ca^2+^ unless calcineurin was also eliminated by either mutations or inhibitors such as cyclosporine and FK506 (Cunningham and Fink 1996). These studies suggest that activated calcineurin may somehow inhibit Vcx1 function, while independently inducing Pmc1 and Pmr1 expression via Crz1 activation (see Figure 1A). However, the molecular mechanism of Vcx1 inhibition by calcineurin has not been elucidated and the interaction could be indirect.

**Figure 1:**
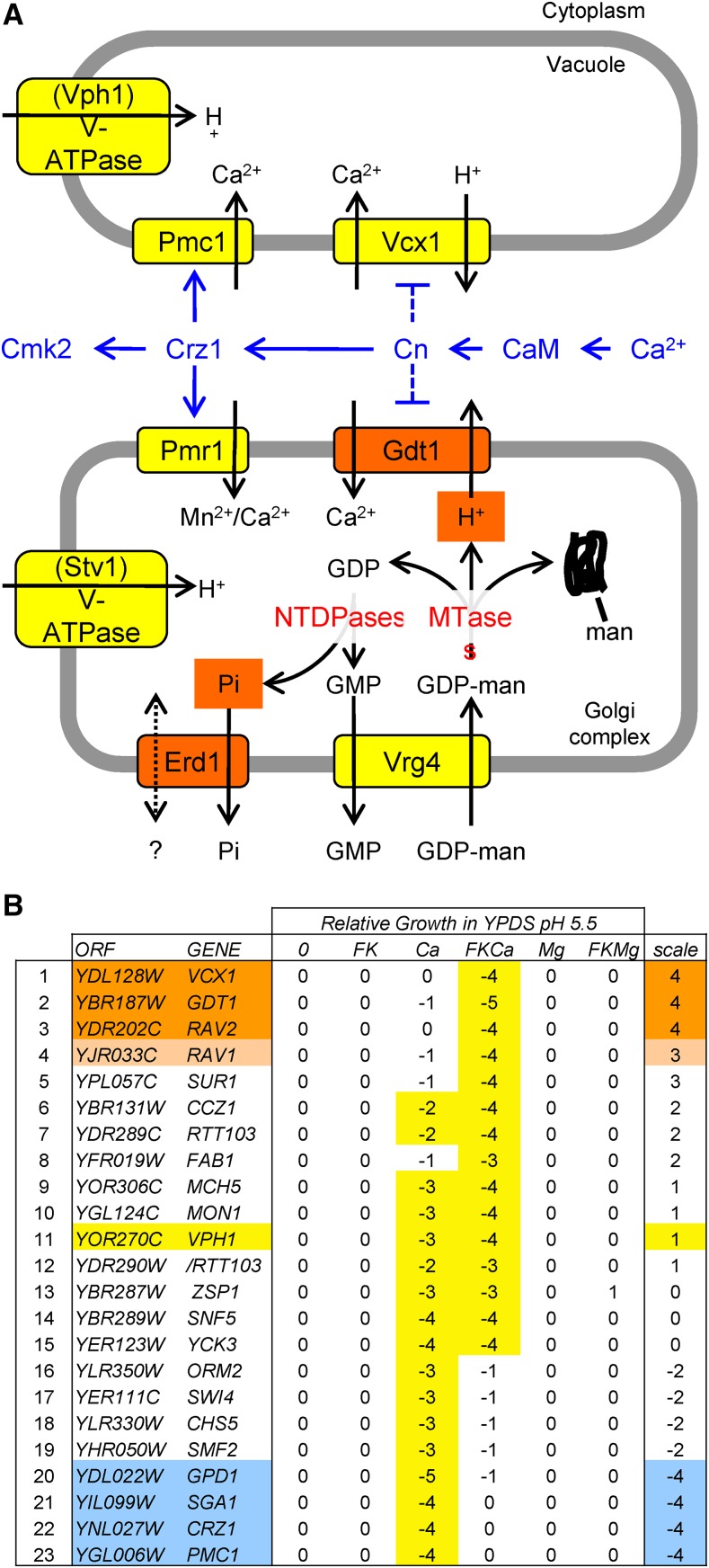
Model of Ca^2+^ homeostasis and Golgi glycosylation, with results from a genome-wide screen. (A) Model of known ion or nucleotide sugar transporters (yellow) in the vacuole and Golgi complex that are studied here along with modes of regulation (blue) by the Crz1 transcription factor, calcineurin, calmodulin, and high cytosolic Ca^2+^. Gdt1 and Erd1 (orange) are putative transporters of the byproducts of glycosylation reactions in the Golgi complex such as H^+^ [produced by mannosyltransferases (MTases)] and Pi [produced by nucleoside triphosphate diphosphatases (NTDPases)] that were identified in genetic screens. (B) Results of a genetic screen for knockout mutants that specifically exhibit hypersensitivity to elevated Ca^2+^ and/or Ca^2+^ plus FK506 in the growth medium. The numbers indicate growth relative to wild-type controls (smaller numbers indicate slower growth) with the strongest effects highlighted (yellow). The 23 filtered mutants were ranked from *vcx1∆*-like (orange) to *pmc1∆*-like (blue) based on the difference between the two Ca^2+^ conditions.

Here we rule out the V-ATPase as a necessary intermediate in the inhibition of Vcx1 by calcineurin, and we search for possible intermediaries through a genome-wide screen of the yeast gene knockout collection. While the *gdt1∆* mutant closely resembled the *vcx1∆* mutant in its hypersensitivity to Ca^2+^ when calcineurin was inhibited or mutated, calcineurin-dependent inhibition of Vcx1 still occurred in *gdt1∆* mutants. Gdt1 localizes to the Golgi complex of yeast, promotes glycosylation in high Ca^2+^ conditions, and transports Ca^2+^ when expressed in heterologous systems (Demaegd *et al.* 2013; Colinet *et al.* 2016; Potelle *et al.* 2016). The sole human ortholog of Gdt1, termed TMEM165, was previously shown to regulate pH and glycosylation in the Golgi complex and to be deficient in individuals with a congenital disorder of glycosylation (Foulquier *et al.* 2012; Zeevaert *et al.* 2013; Potelle *et al.* 2016). Below we show that Gdt1 and Vcx1 both promote Ca^2+^ sequestration when their organelles are properly acidified by the V-ATPase, and both promote Ca^2+^ increases in the cytoplasm when acidification has been disrupted.

To investigate how calcineurin might regulate Gdt1 function, we isolated spontaneous mutations that exhibited elevated Gdt1 function even while calcineurin remained functional. Mutants deficient in Erd1, a polytopic transmembrane protein important for glycosylation and sorting of proteins in the Golgi complex (Hardwick *et al.* 1990), were recovered. We show evidence that Erd1 recycles Pi byproducts of the glycosylation in the Golgi complex before this important nutrient is lost by exocytosis. Therefore, this study sheds new light on the mechanisms that sustain luminal glycosylation reactions in the Golgi complex and promote Pi, Ca^2+^, and H^+^ homeostasis in the cell.

## Materials and Methods

### Yeast strains and genetic screens

The yeast knockout collection in strain BY4741 background (Giaever *et al.* 1999) was inoculated into 200 µl yeast extract peptone dextrose (YPD) medium in 96-well dishes and grown to stationary phase (2 d incubation at 30°). Dishes were vortexed briefly to suspend the settled cells, then pinned onto noble agar medium containing YPD medium plus 5 mM succinic acid and supplemented with either 200 mM CaCl_2_, 200 mM MgCl_2_, 1 µg/ml FK506, or combinations thereof. After incubation for 3 d at 30°, each strain was scored manually for growth relative to wild-type controls that were included in the arrays, using a scale ranging from 2 (increased growth) through 0 (wild-type growth) to −5 (greatly decreased or no growth). The screen was repeated twice independently. Of the 107 mutant strains that consistently exhibited a significant response to at least one condition, 84 were found to exhibit elevated Ca^2+^ influx in YPD medium or YPD medium plus FK506 (Martin *et al.* 2011). The remaining 23 mutants contained *vcx1∆* and *pmc1∆* and were ranked on a scale from *vcx1∆-like* to *pmc1∆-like* based on their distinct behaviors in media containing Ca^2+^ and Ca^2+^ plus FK506. A *gdt1∆* mutation was generated in a W303-1A strain background and crossed with strains bearing *vcx1∆*, *pmc1∆*, *crz1∆*, and *pmr1∆* to produce a panel of isogenic strains bearing many combinations of these mutations in both mating types. Genotypes were confirmed by marker analyses and by polymerase chain reaction (PCR) confirmations.

To identify spontaneous mutants that increase Ca^2+^ tolerance of *crz1∆ pmc1∆ vcx1∆* triple mutants, two strains of opposite mating types and with different selectable markers (DDY19 and K1357) were streaked for single colonies on agar YPD medium, and 36 single colonies were picked, grown further on the same medium to allow spontaneous mutations to accumulate, and then incubated on noble agar YPD medium containing 5 mM succinic acid and 111 mM CaCl_2_ for several days. A single Ca^2+^-tolerant colony was picked in each case, purified by restreaking, and then mated with one another in all possible combinations. The resulting diploids were reanalyzed for Ca^2+^ tolerance. This complementation test indicated that 26 haploid strains contained dominant mutations in unknown genes and 10 haploid strains contained recessive mutations all in the same gene. The 10 recessive mutants were fortuitously found to exhibit hypersensitivity to tunicamycin. One recessive mutant was transformed with a low-copy plasmid library containing fragments of yeast genomic DNA, and nine transformants were replica plated to YPD plus 2 µg/ml tunicamycin to select for strains bearing complementing plasmids. Two different plasmids overlapping the *ERD1* gene and several nearby genes were recovered and found to reverse both the tunicamycin hypersensitivity and the Ca^2+^ tolerance phenotypes upon retransformation into the recessive mutant strains. *ERD1* was identified as the defective gene in the recessive mutants by (1) introducing an *erd1∆* null mutation into the K1357 strain and obtaining both the tunicamycin hypersensitivity and the Ca^2+^ resistance phenotypes and (2) demonstrating non-complementation between the *erd1∆* null strain and the spontaneous recessive mutant strains. Similar *erd1∆* knockout mutations were also introduced by transformation into several other strains; all strains utilized in figures are listed in Supplemental Material, Table S2. Table S1 lists additional strains in the W303 background, their genotypes, and quantitative analyses of their functions in Ca^2+^ tolerance.

### Ion tolerance and Pi dependence assays

Yeast strains were grown to saturation at 30°, typically overnight, in YPD medium for ion tolerance assays or in synthetic complete (SC) medium for Pi dependence assays. They were then diluted 1:1000 into fresh media containing 5 mM succinic acid and various concentrations of CaCl_2_ in 96-well dishes with and without 1 µg/ml FK506, mixed, and incubated at 30° for 24 hr without shaking. The cells were resuspended by vortex mixing and optical density was measured at 650 nm using a microplate spectrophotometer (Molecular Devices). The concentration of Ca^2+^ causing a 50% decrease in maximal optical density (the IC50) and the concentration of Pi permitting growth to 50% maximal optical density (the ED50) were calculated for each strain by non-linear regression using the sigmoid equation with four parameters (maximum OD650, minimum OD650, IC50 or ED50, and slope factor). The averages from two independently generated strains of the same genotype (±SD) were calculated and plotted. In Table S1, the coefficient of variation (CV) is listed instead of SD, and the data from two separate experiments (A and B) were normalized to a third reference dataset using linear regression after log-transformation.

### PMC1-lacZ expression assays

The *PMC1-lacZ* reporter gene carried on the high-copy plasmid pKC190 (Cunningham and Fink 1996) was transformed into the indicated strains, and three independent transformants were grown to log phase in SC-ura medium, pelleted briefly, and suspended in YPD medium with 5 mM succinic acid containing 0, 50, or 100 mM CaCl_2_. After 4 hr incubation at 30°, cells were pelleted, resuspended in Z-buffer, permeabilized with sodium dodecyl sulfate (SDS) and chloroform, and assayed for β-galactosidase activity using o-nitrophenyl-β-galactoside as substrate as described previously (Cunningham and Fink 1996).

### Pi export assays

Yeast strains were grown overnight to saturation in SC medium (7.35 mM Pi) and then diluted 1000-fold into fresh SC medium containing tracer quantities (∼30 µCi per ml) of fresh [32]Pi (Perkin Elmer). After 12 hr of incubation with shaking, the log-phase cells were pelleted and washed five times with 5 ml of ice-cold Pi-free SC media to remove all unincorporated [32]Pi from the medium. The cells were then resuspended in Pi-free SC media and incubated on ice or at 30° and sampled at various times. After centrifugation at 15,000 rpm for 1 min, the cell-free supernatant was analyzed by liquid scintillation counting and by thin-layer chromatography (TLC), by spotting 3 μl of sample supernatant onto polyethylenimine (PEI)-cellulose TLC plates, which was then developed in 1 M LiCl with 10 mM HEPES, pH 7.5. The TLC plate was imaged by autoradiography.

### Antibodies and western blotting

Cells were lysed via fast alkaline lysis in 0.5 M NaOH with 1.85% beta-mercaptoethanol (BME) in ice water for 10 min. The protein was then isolated through precipitation by addition of trichloroacetic acid (TCA) to a final concentration of 10% (w/w). Equal volumes of protein isolate and 2× SDS sample buffer (0.1 M Tris-HCl, pH 6.8, 4% SDS, 0.2% bromophenol blue, 20% glycerol, 2% BME) were then combined, and the samples were incubated at 37° for 30 min. Proteins were processed by separation on a 10% SDS/polyacrylamide gel electrophoresis (PAGE) gel and western blotting, as described previously (Mehta 2009). Blots were probed with anti-TAP polyclonal antibodies from rabbit at 1:10,000 dilution (ThermoFisher, CAB1001) or anti-FLAG polyclonal antibodies from rabbit at 1:10,000 dilution (F7425; Sigma, St. Louis, MO). Protein standards were probed with anti-glucose-6-phosphate dehydrogenase polyclonal antibodies from rabbit at 1:10,000 dilution (A9521; Sigma), or anti-tubulin monoclonal antibodies at 1:10,000 dilution (EMD Millipore, MAB1864).

### Statistical tests of significance

Student’s *t*-tests were implemented on many datasets, as indicated in the figures (* *P* < 0.05, ** *P* < 0.01, *** *P* < 0.001).

### Data availability and reagent availability

All yeast strains and plasmids are archived and available upon request. Raw data files used to generate figures and tables are also archived and available upon request. All requests should be made to the corresponding author.

## Results

### Genetic screen for mutants with altered Ca^2+^ sensitivity: V-ATPase, Vcx1, and Gdt1

The ability of yeast cells to proliferate in high Ca^2+^ environments depends mostly on the vacuolar Ca^2+^ ATPase (Pmc1) when calcineurin is functioning, and mostly on the vacuolar H^+^/Ca^2+^ exchanger (Vcx1) when calcineurin has been inhibited or mutated (Cunningham and Fink 1996). To search for additional Ca^2+^ transporters or regulators of Vcx1, we screened a collection of 4848 non-essential gene knockout mutants of yeast strain BY4741 for their ability to proliferate in media containing 200 mM CaCl_2_ with and without the calcineurin inhibitor FK506 (see *Materials and Methods*). A similar genetic screen using cyclosporine instead of FK506 to inhibit calcineurin yielded partially overlapping results (Zhao *et al.* 2013). After filtering all the mutants that were hypersensitive to FK506 alone or to control osmolyte (200 mM MgCl_2_), and then filtering out the candidates that had exhibited elevated Ca^2+^ uptake in response to FK506 in a previous study (Martin *et al.* 2011), a total of 23 mutants were hypersensitive to Ca^2+^, Ca^2+^ plus FK506, or both while passing the stringent filters. These 23 mutants were ranked on a scale ranging from “*vcx1∆*-like” to “*pmc1∆*-like” (Figure 1B). As expected, the *crz1∆* mutant ranked closest to the *pmc1∆* mutant because the calcineurin-dependent transcription activator Crz1 strongly induces expression of Pmc1 (Matheos *et al.* 1997; Stathopoulos and Cyert 1997). Two other mutants (*gpd1∆*, *sga1∆*) exhibited hypersensitivity to Ca^2+^ but not Ca^2+^ plus FK506, but were not studied further.

The three mutants closest to *vcx1* (*gdt1∆*, *rav1∆*, *rav2∆*) may be deficient in positive regulators of Vcx1 or in novel Ca^2+^ transporters. Rav2 forms a complex with Rav1 and promotes assembly of V1 and V0 sectors of the vacuolar form of the V-ATPase (Smardon *et al.* 2014). The low residual vacuolar V-ATPase activity present in *rav1∆* and *rav2∆* mutants may be insufficient to power the H^+^/Ca^2+^ exchange activity of Vcx1, thereby producing the *vcx1∆*-like phenotype. Complete loss of all V-ATPase forms, as observed in *vma1∆* and other *vma* mutants, resulted in hypersensitivity to high Ca^2+^ and Ca^2+^ plus FK506, but these mutants were filtered out because of hypersensitivity to high Mg^2+^ and Mg^2+^ plus FK506. Surprisingly, a mutant that specifically lacks only the vacuolar form of the V-ATPase (*vph1∆*) while preserving the Golgi/endosomal forms exhibited no hypersensitivity to high Mg^2+^ and strong hypersensitivity to high Ca^2+^ and high Ca^2+^ plus FK506 conditions (Figure 1, line 11), as if the functions of Vcx1 and Pmc1 were both compromised.

**Figure 2:**
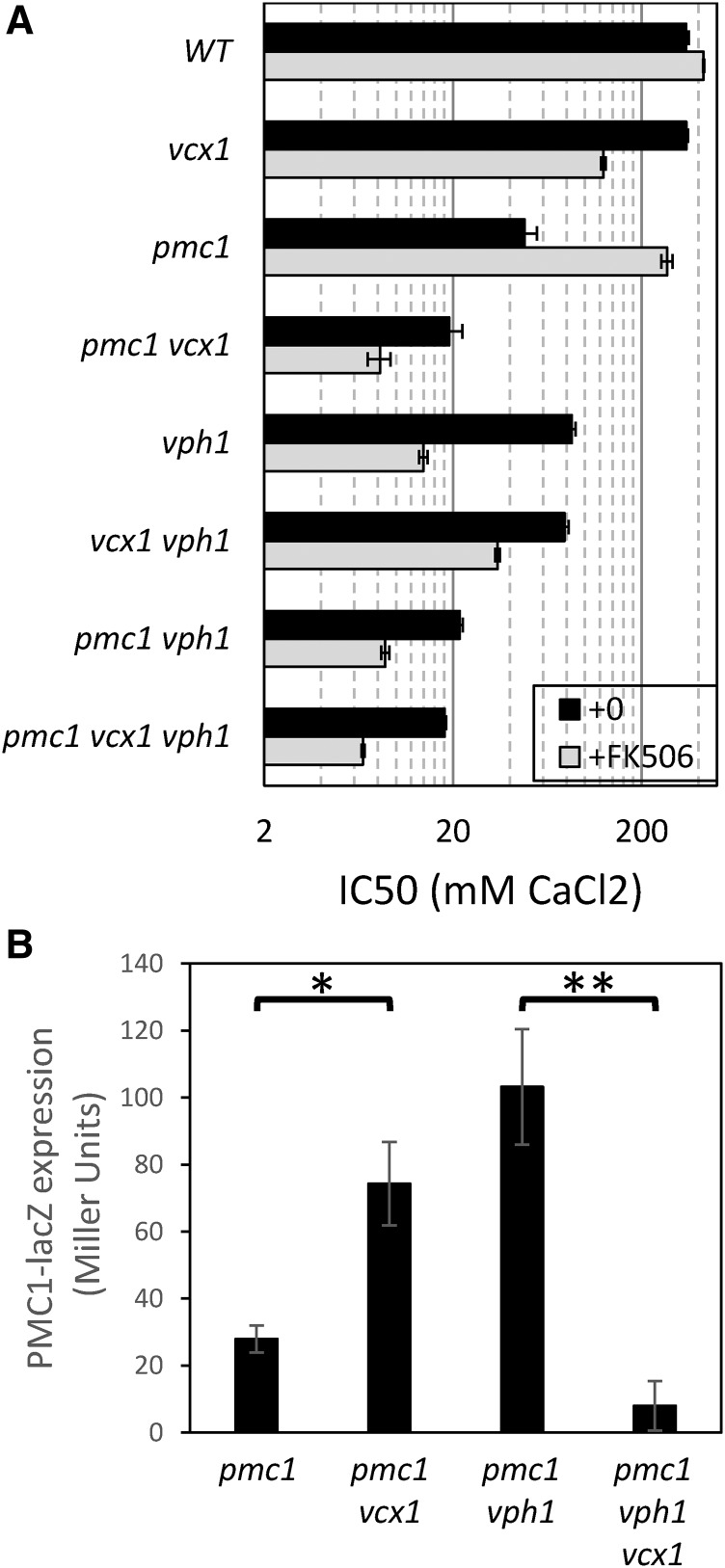
Vacuolar V-ATPase determines directionality of Vcx1 operation. (A) Ca^2+^ tolerance assays were performed in duplicate in YPDS medium (black bars) or in the same medium containing 0.2 µg/ml FK506 (gray bars) as described in *Materials and Methods*, and the derived IC50 values (±SD) for the indicated mutants were plotted on a log scale. (B) The indicated strains were transformed with plasmid pKC190 that bears the *PMC1-lacZ* reporter gene (Cunningham and Fink 1996) and β-galactosidase activity was measured 4 hr after log-phase cells were shifted to YPDS medium containing 100 mM CaCl_2_. Results from three independent transformants were averaged (±SD). * *P* < 0.05, ** *P* < 0.01.

To investigate how the vacuolar V-ATPase affects Pmc1 and Vcx1 function, a *pmc1∆ vcx1∆ vph1∆* triple knockout mutant was constructed in the W303-1A strain background along with all possible double and single knockout mutants, and the concentrations of CaCl_2_ that cause a 50% inhibition of total growth (*i.e.*, the IC50) were compared for all eight strains. Relative to the wild-type parent, the *pmc1∆ vcx1∆ vph1∆* triple mutant was ∼19-fold more sensitive to Ca^2+^ in the medium (Figure 2A, black bars). In the absence of Vph1, restoring Pmc1 increased the IC50 for Ca^2+^ strongly (4.2-fold), whereas restoring Vcx1 had little effect (1.1-fold) in the presence or absence of Pmc1. Restoring Vph1 function had no significant effect in the cells that lack both Pmc1 and Vcx1 (1.05-fold), but had strong effects on cells expressing only Vcx1 (2.2-fold) or Pmc1 (4.4-fold). Therefore, Pmc1 activity was partially dependent on a functional V-ATPase in the vacuolar membrane, and the relatively low Vcx1 activity detectable in *pmc1∆* mutant backgrounds was totally dependent on the vacuolar V-ATPase.

To test whether the vacuolar V-ATPase mediates the interaction between calcineurin and Vcx1, the same eight mutant strains were reanalyzed in the presence of FK506 (Figure 2A, gray bars). The *pmc1∆ vcx1∆ vph1∆* triple mutant became 2.7-fold more sensitive to Ca^2+^ in the presence of FK506 than in the absence of FK506, presumably owing to diminished function of the Crz1 transcription factor and therefore diminished expression of the Pmr1 Ca^2+^/Mn^2+^ ATPase in the Golgi complex when calcineurin is inhibited. Restoring Vcx1 function alone had little effect (1.3-fold increase) in the presence of FK506, whereas restoring Pmc1 function alone had strong effects (5.2-fold increase) despite a lower expression of Pmc1 in the absence of calcineurin (Cunningham and Fink 1994). Importantly, restoring Vcx1 in the *vph1∆* mutant actually *lowered* tolerance to environmental Ca^2+^ by a significant degree (2.5-fold decrease) in the absence of both calcineurin and only when Pmc1 was functioning. All these findings were reproducible in a replicate experiments and show, for the first time, that Vcx1 can antagonize the function of Pmc1 when the vacuoles lack acidification by the V-ATPase and when the cytoplasm lacks calcineurin signaling. Such antagonism was expected, because H^+^/Ca^2+^ exchangers are generally thought to operate in “reverse mode” in conditions where the luminal H^+^ concentration is low and the Ca^2+^ concentration is high, potentially causing futile cycles between Vcx1 and Pmc1 in the absence of Vph1. Reverse-mode operation of Na^+^/Ca^2+^ exchangers is well established (Harper and Sage 2016). That reverse-mode operation of Vcx1 (observed in *vph1∆* mutants with FK506) did not occur in the absence of FK506 suggests that calcineurin regulates both forward and reverse modes of Vcx1 through a process that is independent of the V-ATPase.

Reverse-mode operation of Vcx1 is predicted to increase cytoplasmic free Ca^2+^ concentrations and enhance calcineurin signaling, whereas forward-mode operation achieves the opposite effects. To test this prediction, we measured expression of a calcineurin-sensitive reporter gene *PMC1-lacZ* in *vcx1∆ vph1∆* double mutants relative to the single mutants and control strain. This experiment was performed in a *pmc1∆* mutant background to improve the sensitivity of the reporter and enable detection of forward-mode Vcx1 activity (Cunningham and Fink 1996). As predicted, the *pmc1∆ vcx1∆* strain expressed *PMC1-lacZ* at significantly higher levels than the *pmc1∆* strain after exposure to 100 mM CaCl_2_, whereas the *pmc1∆ vph1∆ vcx1∆* triple mutant strain exhibited much lower levels of expression than the *pmc1∆ vcx1∆* double mutant strain (Figure 2B). These findings show that Vcx1 can lower calcineurin signaling when the vacuole is properly acidified and increase calcineurin signaling when the vacuolar V-ATPase is inactivated, thus providing independent evidence that Vcx1 transports Ca^2+^ bidirectionally, similar to Na^+^/Ca^2+^ exchangers in animals.

### Calcineurin regulates independent functions of Gdt1 and Vcx1

**Figure 3:**
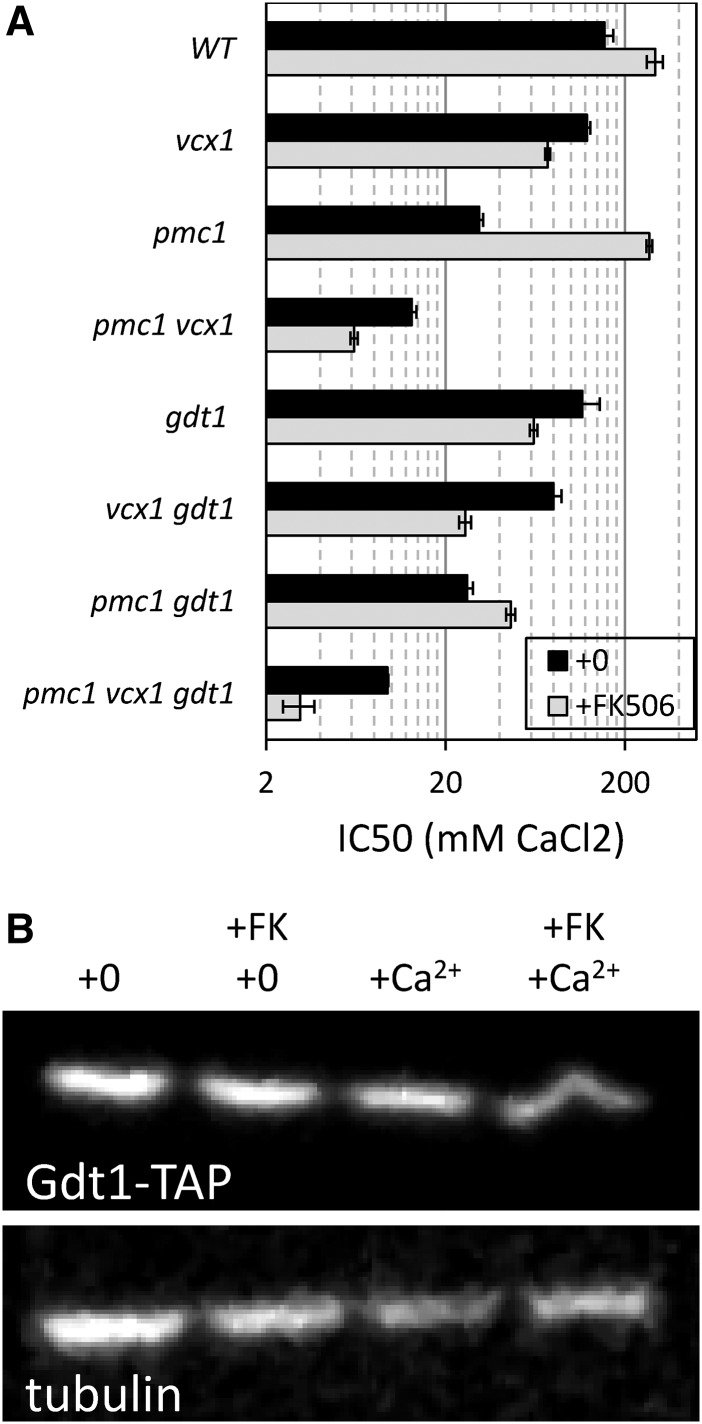
Gdt1 promotes Ca^2+^ detoxification independent of Vcx1 and Pmc1. (A) A panel of *Saccharomyces cerevisiae* strains lacking Gdt1, Vcx1, and Pmc1 in all possible combinations was assayed for Ca^2+^ tolerance in the presence (gray bars) or absence (black bars) of FK506 as described in Figure 2A. (B) A strain expressing epitope-tagged Gdt1-TAP was grown to log phase in YPDS medium containing 200 mM CaCl_2_ and/or 1 µg/ml FK506, harvested, lysed, and analyzed by SDS-PAGE and western blotting using anti-TAP polyclonal (top) and anti-tubulin monoclonal (bottom) antibodies.

The *gdt1∆* mutant, which lacks a probable Golgi-localized H^+^/Ca^2+^ exchanger (Demaegd *et al.* 2013; Colinet *et al.* 2016), clustered closest to the *vcx1∆* mutant in our screening conditions (Figure 1B) and therefore is a potential regulator of Vcx1. To test whether Gdt1 promotes Ca^2+^ resistance independent of Vcx1, the *gdt1∆* knockout mutation was introduced into the *pmc1∆ vcx1∆* double mutant and the single mutants in the W303-1A background, and the IC50s of Ca^2+^ were quantified in the presence and absence of FK506. The *gdt1∆* mutation weakly diminished Ca^2+^ tolerance in every strain background when calcineurin was functional (average decline of 1.35 ± 0.15 fold) but strongly diminished Ca^2+^ tolerance in WT, *pmc1∆*, *vcx1∆*, and *pmc1∆ vcx1∆* backgrounds when FK506 was present (by 4.8, 2.9, 5.9, and 2.0 fold, respectively; Figure 3A). Similar results were obtained in four replicate experiments and when calcineurin was inactivated by a *cnb1∆* knockout mutation (data not shown). Thus, in the absence of calcineurin signaling, Gdt1 strongly promoted Ca^2+^ tolerance independent of both Vcx1 and Pmc1, and likely represents a new class of Ca^2+^ transporter that is partially inhibited by calcineurin.

Calcineurin may directly or indirectly regulate Gdt1 function. To test whether calcineurin inhibits Gdt1 function through its ability to activate Crz1 or to induce expression of Cmk2, we repeated the Ca^2+^ tolerance experiments described above in *crz1∆ cmk2∆* double mutant and single mutant backgrounds. This experiment ruled out Crz1 and Cmk2 as possible intermediaries in the inhibition of Gdt1 by calcineurin (see Table S1). To test whether calcineurin signaling could diminish expression of Gdt1 or alter its mobility on SDS-PAGE, western blots were performed on cells containing a tandem affinity purification (TAP) epitope tag, integrated at the 3′ end of the chromosomal *GDT1* gene (Ghaemmaghami *et al.* 2003), which were exposed to Ca^2+^ with and without FK506. The TAP tag did not alter the Ca^2+^ tolerance functions of Gdt1 or its responsiveness to FK506 (not shown). After exposure of the cells to 200 mM CaCl_2_ with or without 1 µg/ml FK506 for 12 hr, the Gdt1-TAP band maintained the same intensity and migration on the gel (Figure 3B). Therefore, the inhibitory effects of calcineurin on Gdt1 function were not noticeably associated with changes in Gdt1 expression or modification, and are possibly dependent on unknown intermediaries.

### Gdt1 promotes H^+^/Ca^2+^ exchange in the Golgi complex

**Figure 4:**
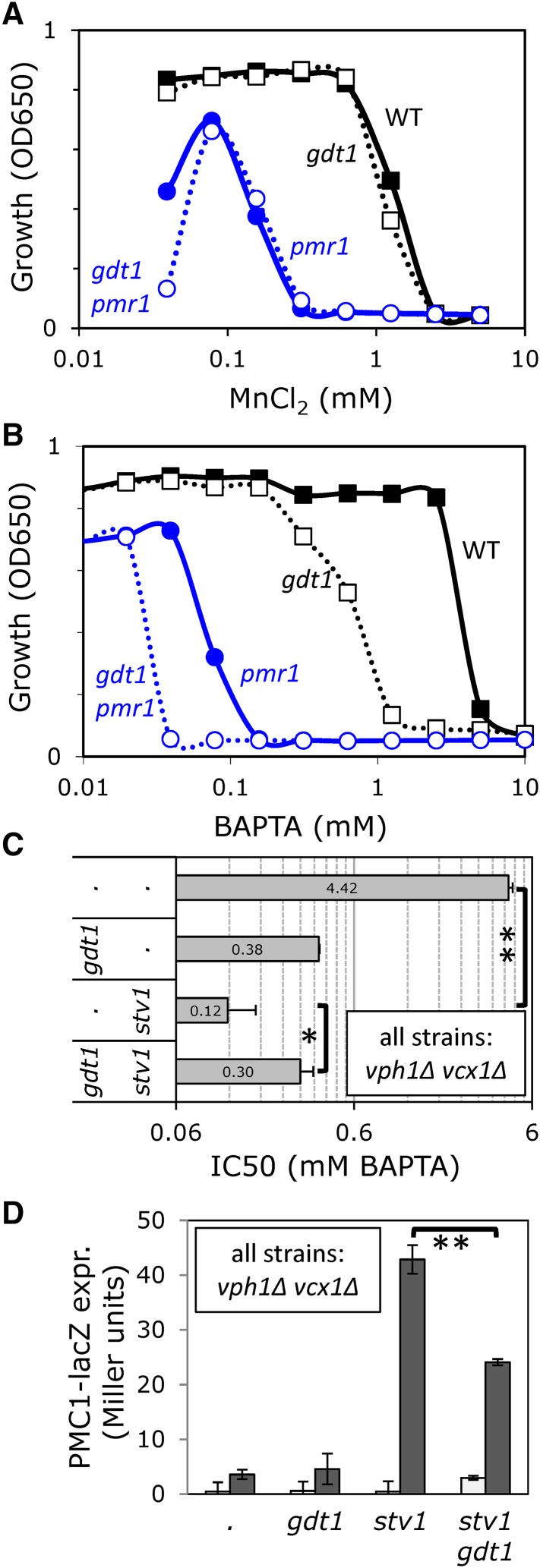
Gdt1 supplies essential Ca^2+^ independent of Pmr1, and reverse-mode activity of Gdt1 is blocked by Golgi V-ATPase. Mn^2+^ (A) and BAPTA (B and C) tolerance assays were performed in YPD medium, and raw data (A and B) or derived IC50 values (C; ±SD) were plotted on log scales. (D) Expression of *PMC1-lacZ* reporter gene was measured as described in Figure 2B except the medium contained either 0 mM (white bars) or 50 mM (gray bars) supplemental CaCl_2_. * *P* < 0.05, ** *P* < 0.01.

Recent studies also showed that Gdt1 localizes to the Golgi complex of yeast (Demaegd *et al.* 2013), and we have independently confirmed those findings using immunofluorescence microscopy and sucrose gradient fractionation of a functional Gdt1-3HA fusion protein (data not shown). This finding led us to explore the possible interactions between Gdt1 and Pmr1, the secretory pathway Ca^2+^ ATPase of yeast. To determine whether Gdt1 supplies essential Ca^2+^ or Mn^2+^ to the Golgi complex independent of Pmr1, we quantified the tolerance of *gdt1∆ pmr1∆* double mutant and single mutant strains to either high Mn^2+^ (Figure 4A) or a membrane-impermeant chelator of Ca^2+^, Mn^2+^, and other divalent cations (BAPTA, Figure 4B) in the culture medium. The Mn^2+^ tolerance of *gdt1∆* mutants and *gdt1∆ pmr1∆* double mutants were indistinguishable from control strains (wild type and *pmr1∆*, respectively), suggesting that Gdt1 cannot remove toxic Mn^2+^ from the cytoplasm. The *gdt1∆* mutant exhibited a strong hypersensitivity to BAPTA relative to the wild-type parent strain, and the *gdt1∆ pmr1∆* double mutant exhibited extreme hypersensitivity to BAPTA that was significantly greater than the *pmr1∆* mutant (Figure 4B). Thus, Gdt1 appeared to supply essential Ca^2+^ ions to the Golgi complex independent of Pmr1.

Interestingly, FK506 blocked growth of *gdt1∆ pmr1∆* double mutants in all growth media we tested (data not shown). This lethality of FK506 suggests that calcineurin-dependent upregulation of Pmc1 and relocalization to the Golgi complex (Marchi *et al.* 1999) is crucial to supply essential Ca^2+^ when Pmr1 and Gdt1 are absent, but further experiments are required to test this hypothesis.

If Gdt1 functions as a H^+^/Ca^2+^ exchanger in the Golgi, its contributions to BAPTA tolerance should depend on the Golgi/endosomal forms of the V-ATPase, which employ Stv1 rather than Vph1 in the V0-sector of the enzyme (Manolson *et al.* 1994). However, *stv1∆* mutants alone behaved like wild type in all conditions tested, because the remaining Vph1-containing V-ATPase fully acidifies the Golgi even in the absence of Stv1 (Qi and Forgac 2007). We therefore tested the BAPTA tolerance of *gdt1∆* and *stv1∆* mutations in a background that lacks Vph1 (and also Vcx1, which could complicate the analyses). In this *vph1∆ vcx1∆* double mutant background, the loss of Gdt1 caused a more than 10-fold decrease in BAPTA tolerance and the further loss of Stv1 had little additional effect (Figure 4C). The loss of Stv1 alone caused a more than 25-fold decrease in BAPTA tolerance, and this hypersensitivity was partially reversed (∼2.5-fold) by the further loss of Gdt1. Thus, like Vcx1, Gdt1 appeared to function in reverse mode when the V-ATPase was completely eliminated and in forward mode when the V-ATPase was functional.

The ability of Gdt1 to decrease and increase cytosolic free Ca^2+^ was also examined by measuring expression of the calcineurin-dependent *PMC1-lacZ* reporter gene described earlier. In the *vph1∆ vcx1∆* double mutant background that acidifies the Golgi complex, the additional *gdt1∆* mutation did not significantly alter expression of the reporter gene after addition of 50 mM CaCl_2_ (Figure 4D), and so forward-mode activity of Gdt1 was not detectable above the high influences of Pmc1 and Pmr1 in these conditions. However, reverse-mode activity of Gdt1 was detected in the *vph1∆ vcx1∆ stv1∆* triple mutant background, because the additional *gdt1∆* mutation significantly lowered expression of the reporter gene (Figure 4D). Together with the earlier findings on Gdt1 and Vcx1 bidirectionality, these findings provide strong support for the hypothesis that Gdt1 functions as a reversible H^+^/Ca^2+^ exchanger of the Golgi complex *in vivo*.

### Erd1 recycles inorganic phosphate from the ER and Golgi complex and limits the functions of Pmr1 and Gdt1

If activated calcineurin inhibits Gdt1 function, it may be possible to isolate variants of Gdt1 that are hyperactive when calcineurin is fully functional, similar to the hyperactive Vcx1-D mutants that have been isolated previously (Cunningham and Fink 1996). To focus on mutants that disrupt the interaction between calcineurin and Gdt1, we generated strains simultaneously lacking Pmc1, Vcx1, and Crz1, and selected for rare spontaneous mutants that enabled growth in medium supplemented with high concentrations of CaCl_2_ (see *Materials and Methods*). Of 36 independent spontaneous mutations, 26 were found to be dominant in heterozygous diploids. The *GDT1* coding sequence from all 26 strains was amplified by PCR and sequenced. No mutations were identified in the *GDT1* coding sequences. The mechanism of Gdt1 regulation by calcineurin remains unknown and may involve several unknown intermediary steps (see *Discussion*).

In addition to the 26 dominant mutants, 10 independent recessive mutants were recovered, and these mutants defined a single complementation group. All of the recessive Ca^2+^-resistant mutants were found to be hypersensitive to tunicamycin, an inhibitor of N-glycosylation reactions in the ER (Lehle and Tanner 1976), and we isolated two low-copy plasmids from a library of random genomic DNA fragments that complemented this phenotype. The *ERD1* gene was found to be necessary and sufficient for complementation of both phenotypes, and the *erd1∆* knockout mutation was found to recapitulate both phenotypes in the starting strain background. Though the precise function of Erd1 has not yet been determined, earlier studies show that Erd1 is a polytopic membrane protein important for two Golgi-localized processes: glycosylation of secretory proteins, and retrieval of escaped HDEL-containing proteins back to the ER (Hardwick *et al.* 1990). To determine its role in Ca^2+^ homeostasis, the *erd1∆* mutation was introduced into backgrounds that also lacked Pmc1, Vcx1, Gdt1, and Crz1, and the IC50s for CaCl_2_ were measured as before. These experiments showed that Erd1 significantly decreased the Ca^2+^ tolerance of the *pmc1∆ vcx1∆ crz1∆* triple mutant (1.9-fold decrease), and that this effect was abolished if Gdt1 were eliminated (Table S1). Erd1 actually increased Ca^2+^ tolerance or was neutral in most other conditions. These results are consistent with models where Erd1 selectively diminishes the forward function of Gdt1 in the Golgi complex.

Erd1 contains an EXS domain similar to that of human XPR1 and plant PHO1 proteins, which recently have been shown to export Pi from the cell (Hamburger *et al.* 2002; Giovannini *et al.* 2013). Because Pi is produced in the lumen of the Golgi complex and ER as a byproduct of glycosylation reactions (Figure 1A), it is possible that *erd1∆* mutants fail to recycle Pi to the cytoplasm, and the backlog of Pi in the lumen increases the buffer capacity for Ca^2+^ while also interfering with normal glycosylation and sorting reactions. To test the hypothesis that Erd1 recycles the Pi byproduct of glycosylation from the lumen of secretory organelles, several experiments were performed.

**Figure 5:**
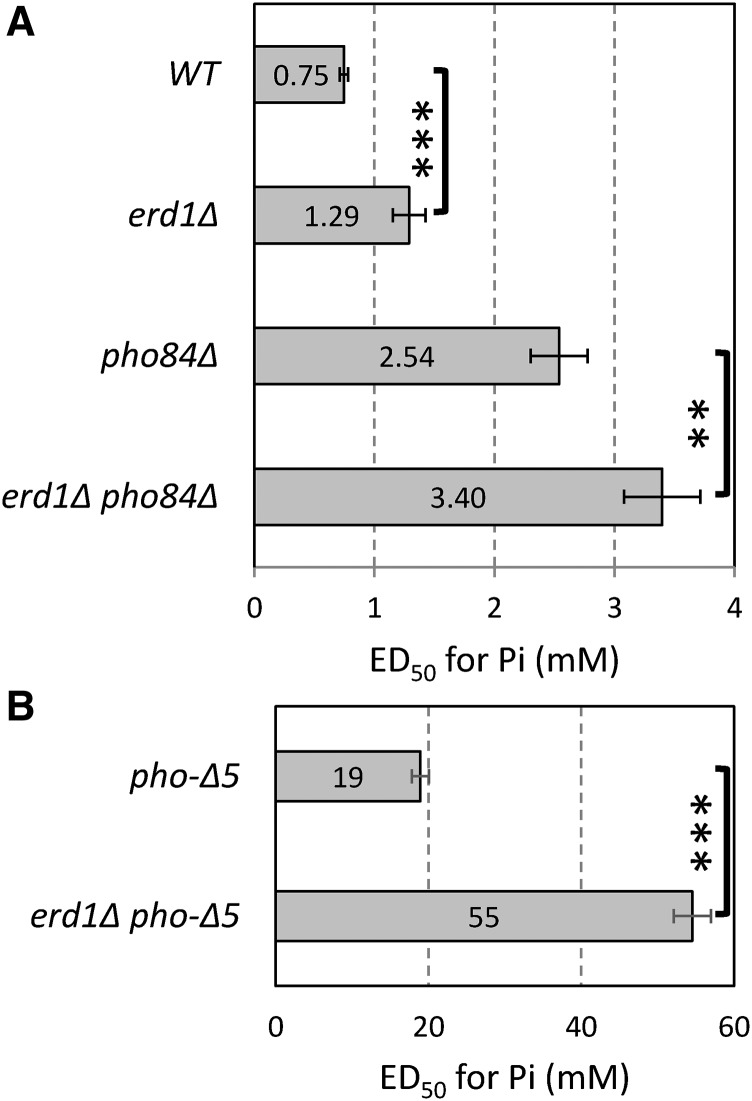
Erd1 supplies essential Pi. The indicated mutant strains were washed and inoculated into an SC medium containing various concentrations of Pi. After 24 hr incubation, the concentration of Pi that enabled 50% maximal growth (*i.e.*, ED50) was derived and the averages (±SD) of triplicate measurements were plotted. The *erd1∆* mutation increased the ED50 by 1.5-fold, 1.4-fold, and 2.9-fold, respectively, in the wild-type (WT), *pho84∆*, and *pho-∆5* (*pho84∆ pho87∆ pho89∆ pho90∆ pho91∆*) backgrounds. ** *P* < 0.01, *** *P* < 0.001. Note the 15-fold change of scale between pho-∆5 strains (B) and other strains (A).

First, because Pi is an important nutrient to yeast, we explored the possibility that Erd1 is important for growth in low-Pi environments. Interestingly, the concentration of Pi in the medium required for 50% maximal growth (*i.e.*, the ED50) was about 1.5-fold higher for *erd1∆* mutants compared with the wild-type control strain (Figure 5A). The *erd1∆* mutation caused a similar increase in the requirement for Pi when introduced into a *pho84∆* mutant background, which lacks a high-affinity Pi transporter in the plasma membrane (Bun-Ya *et al.* 1991) and requires approximately threefold higher concentrations of Pi than wild type for growth (Figure 5A). The *erd1∆* mutation strongly increased the ED50 of Pi nearly threefold when introduced into a *pho84∆ pho87∆ pho89∆ pho90∆ pho91∆* quintuple mutant background (Figure 5B) that lacked all five of the known Pi transporters in yeast (Wykoff and O’Shea 2001; Samyn and Persson 2016). Therefore, Erd1 supplies essential Pi to the cytoplasm of yeast cells through a process that does not rely on any of the known Pi transporters.

**Figure 6:**
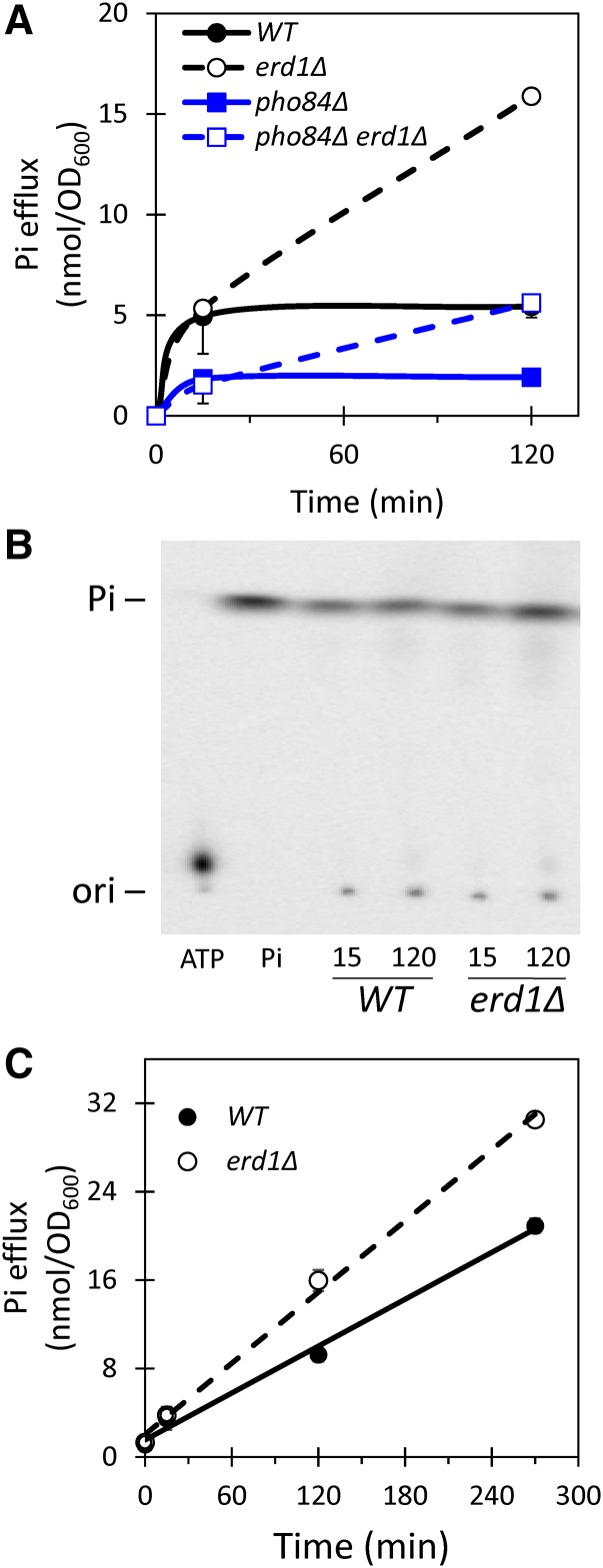
Erd1 prevents Pi loss to the environment. The indicated mutant strains were grown to log phase in SC medium containing tracer levels of [32]Pi radioisotope, washed extensively in Pi-free SC medium, and then in the same medium (A and B) or in SC medium (C). At the indicated times of incubation at 30°, aliquots were removed and cell-free supernatants were analyzed by liquid scintillation counting (A and C) or by TLC (B). Charts illustrate the averages (±SD) of three biological replicates.

**Figure 7:**
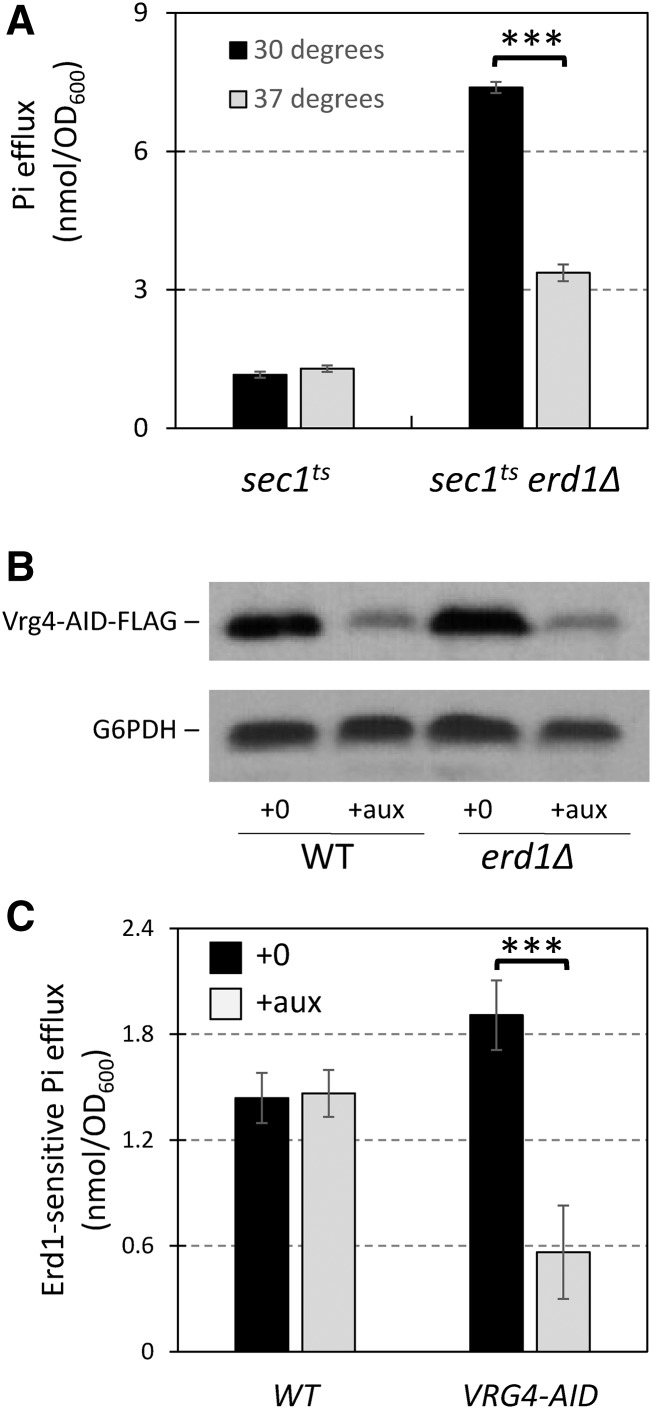
Erd1-sensitive losses of Pi depend on exocytosis and on transport of GDP-mannose into the Golgi complex. (A) Pi losses were measured after 2 hr incubation as in Figure 6A except that *sec1-1^ts^* and *sec1-1^ts^ erd1∆* mutant strains were used at a permissive temperature (30°; black bars) or a non-permissive temperature (37°; gray bars) during the incubation in Pi-free medium. (B) Western blot of Vrg4-AID-FLAG strains after 1 hr exposure to 100 µM auxin in SC medium. (C) Erd1-sensitive losses of Pi (*i.e.*, the difference between *erd1∆* and *ERD1* pairs of strains) were measured for wild-type and Vrg4-AID-FLAG strain backgrounds that were exposed to 100 µM auxin (gray bars) or not (black bars) starting 1 hr before the shifts to Pi-free medium. *** *P* < 0.001.

Second, we tested whether *erd1∆* mutants exhibited higher rates of Pi export into the culture medium via exocytosis, which would be predicted if Erd1 normally recycles this nutrient from the Golgi complex. For this experiment, the *erd1∆* and wild-type strains were cultivated for >10 generations in standard synthetic medium (7.35 mM Pi) supplemented with tracer amounts of [32]Pi, chilled, and the cells were washed extensively in fresh medium lacking Pi. The temperature was then raised to 30° and aliquots were removed at different times, centrifuged to pellet the cells, and the cell-free supernatants were collected and analyzed by liquid scintillation counting and by TLC. In the first 0.25 hr of incubation at 30°, both *erd1∆* and wild-type strains exported similar amounts of radioactivity. However, the *erd1∆* mutant exported strikingly more Pi than wild type over the next 1.75 hr of incubation (Figure 6A, black curves). Similar trends were obtained in the *pho84∆* background (Figure 6A, red curves), though these strains started with <30% of the wild-type levels of Pi owing to their defect in Pi accumulation prior to the start of the experiment. The large majority of radioactivity exported to the culture medium comigrated with Pi standards on TLC (Figure 6B). When the chase experiment was repeated in SC medium containing 7.35 mM Pi, the influx of non-radioactive Pi permits sustained efflux of [32]Pi from wild-type cells for several hours, and still the *erd1∆* mutant exported more [32]Pi (Figure 6C). If this Erd1-sensitive Pi efflux depends on exocytosis, such efflux may be diminished in a *sec1-1^ts^* mutant background, which quickly accumulates post-Golgi secretory vesicles when shifted to the non-permissive temperature of 37° owing to defects in the exocytotic machinery (Novick *et al.* 1981). Remarkably, the Erd1-sensitive efflux of Pi was markedly diminished in the *sec1-1^ts^* strain at 37° but not in the wild-type control strain (Figure 7A). These data suggest that Erd1 normally limits the export of Pi from cells via exocytosis.

If glycosylation reactions in the Golgi complex produce the Pi that is recycled by Erd1, the Erd1-sensitive Pi efflux may be diminished by inactivation of Vrg4, the main nucleotide sugar transporter of the Golgi complex that primarily supplies GDP-mannose in exchange for luminal GMP (Dean *et al.* 1997). Vrg4 is essential for yeast growth and is not inhibited by any known molecules. To render Vrg4 druggable, we introduced an auxin-inducible degron tag at its C-terminus along with a FLAG tag and an expression cassette for osTIR, an auxin-sensitive E3 ubiquitin ligase (Nishimura *et al.* 2009; Morawska and Ulrich 2013). Strains bearing a Vrg4-AID-FLAG fusion protein grew as well as wild type in the absence of auxin, but grew much more slowly than wild type in the presence of 100 µM auxin (data not shown), suggesting that Vrg4 expression became growth-limiting in these conditions. Indeed, just 1 hr exposure to 100 µM auxin resulted in a 70% decline in Vrg4-AID-FLAG expression (Figure 7B). The *erd1∆* mutation was introduced into the Vrg4-AID-FLAG and wild-type control strains, and Erd1-sensitive Pi efflux was measured as above. After a 2 hr chase period in Pi-free culture medium, the Erd1-sensitive Pi efflux from wild-type cells was unaffected by exposure to auxin (Figure 7C). The Erd1-sensitive Pi efflux from the Vrg4-AID-FLAG strain was similar to wild type in the absence of auxin, and was greatly reduced when the cells were exposed to auxin beginning 1 hr before the washes and continuously during the chase period (Figure 7C). These findings show that the GDP-mannose transporter Vrg4 is a major source of the Pi that is lost to the environment in *erd1∆* mutants. Altogether, the results indicate that Erd1 facilitates recycling of the Pi byproduct of glycosylation in the Golgi complex, which alters buffering of Ca^2+^ and performance of Gdt1.

## Discussion

The Golgi complex of all eukaryotes is a hub for glycosylation, sorting, and processing of secreted proteins, transmembrane proteins, ceramides, and other lipids. In yeast, the glycosylation system of the Golgi complex is essential for viability, as mutations that eliminate the major nucleotide-sugar transporter (Vrg4) or the luminal nucleoside triphosphate diphosphatases (Gda1 and Ynd1) are lethal (Dean *et al.* 1997; Gao *et al.* 1999). While the nucleoside monophosphate byproduct of glycosylation is exchanged from the Golgi complex for another nucleotide sugar molecule, the fate of the other byproducts, Pi and H^+^, were elusive until now. Our findings above suggest that Erd1 facilitates transport of the luminal Pi to the cytoplasm where it can be reutilized and that Gdt1 transports luminal H^+^ to the cytoplasm in exchange for cytoplasmic Ca^2+^. Because neither protein has been purified to homogeneity and analyzed in reconstituted liposomes, it remains possible that Erd1 and Gdt1 do not directly transport these byproducts of glycosylation and instead regulate unknown transporters that have these properties. Nevertheless, the simplest model of inorganic ion homeostasis in the Golgi complex (summarized in Figure 1A) is consistent with numerous genetic observations in both yeast and humans, and it also raises several new questions as well as new opportunities for treating a congenital disorder of glycosylation in humans (Dulary *et al.* 2017).

### Pi homeostasis in the Golgi complex

Our findings that Erd1 decreases the loss of Pi from the Golgi complex to the environment and promotes growth of yeast cells in low-Pi environments independent of all known Pi transporters suggest that yeast normally recycles this byproduct of glycosylation for reuse in the cytoplasm. This function is somewhat surprising because XPR1, a homolog of Erd1 in the basolateral plasma membrane of humans, has been shown to promote export of cytoplasmic Pi from the cytoplasm rather than import (Giovannini *et al.* 2013). XPR1 orthologs in zebrafish may have similar roles in Pi export, as knockout mutations resulted in failure to produce osteoclasts (Meireles *et al.* 2014), which are thought to experience massive Pi uptake and efflux during resorption of bone. PHO1, an ortholog of XPR1 in the plant *Arabidopsis thaliana*, has also been shown to promote Pi export from root stelar cells to the xylem, resulting in Pi deficiencies in the shoots (Hamburger *et al.* 2002; Wege and Poirier 2014). PHO1 localizes primarily to the Golgi complex rather than the plasma membrane and thus may export Pi through exocytosis pathways (Arpat *et al.* 2012). The transmembrane topologies of Erd1 (Kim *et al.* 2006) and PHO1 (Wege *et al.* 2016) are similar, so the directionality of net Pi transport by members of the ESX family may depend on other factors such as unknown ions or molecules that could be co- or countertransported with Pi.

Though absent from Erd1, SPX domains are found at the N-termini of XPR1, PHO1, and many other proteins that regulate Pi homeostasis (Secco *et al.* 2012). Deletion of the SPX domain did not diminish Pi transport activity or alter directionality (Wege *et al.* 2016). The SPX domain can also be found at the N-termini of numerous other proteins involved in Pi homeostasis in yeast, such as Pi transporters of the SL13 family (Pho87, Pho90, Pho91), subunits of the vacuolar poly-Pi synthase (Vtc2, Vtc3, Vtc4, Vtc5), a cyclin-dependent kinase (Pho85) and its inhibitor (Pho81) that govern the response to Pi starvation, glycerophosphocholine phosphodiesterase 1 (Gde1), and another ESX domain protein of unknown function (Syg1) (Secco *et al.* 2012). Several SPX domains have now been shown to bind inositol polyphosphates such as IP6 and IP7 (Lee *et al.* 2007; Wild *et al.* 2016), which have important regulatory effects on Pi homeostasis. The lack of an SPX domain in Erd1, and the inability of Pho4 to stimulate transcription of the *ERD1* gene in Pi-limiting conditions, suggest that Erd1 may escape the conventional regulation imposed on other Pi homeostasis regulators.

The evolutionary origins of Erd1 also may be instructive about its functions in cell physiology. Our searches of protein databanks and multiple sequence alignments (not shown) reveal orthologs of Erd1 in virtually all species of fungi, in a unicellular relative of fungi (the nucleariid *Fonticula alba*), and in a unicellular relative of metazoans (the choanoflagellate *Monosiga brevicollis*), but not in metazoans. This suggests that the Erd1 subfamily of ESX-domain proteins originated prior to the divergence of fungi and animals but were uniquely lost from the metazoan lineage. This loss in metazoans could have been enabled by the gain of another Pi transporter in the Golgi complex. Alternatively, the benefits of Pi recycling from the Golgi complex may have diminished in metazoans, where the loss of Pi to the extracellular fluids is not harmful or is potentially even beneficial for Pi storage, for pH buffering, or for creating extracellular structures.

In addition to defects in Pi recycling, *erd1∆* mutants of yeast exhibit striking deficiencies in secretory protein glycosylation and in sorting by the H/KDEL receptor (Erd2), which ordinarily retrieves many ER-resident molecular chaperones and enzymes that have escaped the ER (Hardwick *et al.* 1990). How elevated Pi in the lumen of Golgi complex can cause such disparate effects is not clear. Conceivably, high luminal Pi may competitively inhibit the activities of the NTPDases that produce it, or the glycosyltransferases, nucleotide sugar transporters, and other factors involved directly in promoting glycosylation. The binding of H/KDEL peptides to their receptor is highly sensitive to the luminal pH (Wilson *et al.* 1993), which might be altered in *erd1∆* mutants through accumulation of H_2_PO_4_^−^ and dissociation to H^+^ and HPO_4_^2−^. An inability of *erd1∆* mutants to transport Pi out of the Golgi complex may increase the buffering of Ca^2+^, Mn^2+^, and Mg^2+^ in the lumen as well, perhaps altering the structure of the organelle or decreasing the performance of glycosylation and ER retrieval systems. Lastly, the concentration of some other molecules in the Golgi complex could be directly disrupted in *erd1∆* mutants if Erd1 (or possibly its associated catalytic partner) also co- or countertransports some other molecule together with Pi. Direct transport studies using liposomes with purified and reconstituted Erd1 protein and other biochemical experiments will be needed to test these possibilities.

### Ca^2+^ homeostasis in the Golgi complex and vacuole

Here we show that Gdt1 promotes growth of yeast cells in both low and high Ca^2+^ environments independent of the known Ca^2+^ transporters (Pmr1, Pmc1, Vcx1) and only when the Golgi complex receives sufficient acidification from the V-ATPase. In the absence of all V-ATPase activity, Gdt1 seemed to partially undo the work of the other transporters and to have the opposite effects on cell growth and on calcineurin activation in the cytoplasm. By showing that Gdt1 can operate in reverse mode when the V-ATPase is eliminated, we add strong experimental support to the hypothesis that Gdt1 catalyzes H^+^/Ca^2+^ exchange in the Golgi complex (Demaegd *et al.* 2013; Colinet *et al.* 2016). But unlike Pmr1, which transports both Ca^2+^ and Mn^2+^ to the Golgi complex, we did not detect any hypersensitivity of *gdt1∆* mutants to elevated Mn^2+^ in the medium even in the absence of Pmr1 (data not shown), suggesting that Gdt1 may not transport significant levels of Mn^2+^. Consistent with these findings, the partial rescue of glycosylation defects in *pmr1∆* mutants by supplemental Ca^2+^ depended on Gdt1, but the partial rescue by supplemental Mn^2+^ occurred independent of Gdt1 (Colinet *et al.* 2016). While these findings suggest that Gdt1 does not play a direct part in Mn^2+^ transport into the Golgi complex, direct transport of Mn^2+^ by Gdt1 was proposed to explain the observation that *gdt1∆* mutants exhibit glycosylation defects in high Ca^2+^ conditions that can be rescued by supplemental Mn^2+^ and Pmr1 function (Potelle *et al.* 2016). Our demonstration of reverse-mode operation of Gdt1 strengthens an alternative hypothesis to explain this observation: in high Ca^2+^ conditions, Gdt1 may normally promote net Ca^2+^ efflux from the Golgi complex and thereby allow Pmr1 to cycle more rapidly and effectively transport more Mn^2+^ for stimulation of glycosyltransferases.

Findings from mammalian cells indicate broad conservation of Gdt1 and TMEM165 function in the Golgi complex. Simultaneous knockouts of TMEM165 and SPCA1 genes (a homolog of Pmr1 encoded by *ATP2C1*) result in synthetic lethality in the human HAP1 cell line (Blomen *et al.* 2015), suggesting that these Golgi proteins share important functions. Deficiency of TMEM165 in human cells disrupts pH homeostasis of lysosomes and late endosomes (Demaegd *et al.* 2013), as expected if TMEM165 normally catalyzes H^+^/Ca^2+^ exchange in the forward mode in these cells. Interestingly, knockdown of TMEM165 in human cell lines resulted in glycosylation defects that could be rescued by low concentrations of Mn^2+^ (Potelle *et al.* 2016). Such rescue provides clues for therapies to treat rare deficiencies of TMEM165 in humans, which cause a type-II congenital disorder of glycosylation that manifests with glycosylation defects, bone dysplasias, and other abnormalities (Dulary *et al.* 2017). TMEM165 appears to be expressed in virtually all tissues, so understanding how such specific developmental abnormalities arise from defects in a housekeeping gene function will require much more work. The recent identification of TMEM165 splice variants localized to the ER, rather than the Golgi, raises the possibility of additional functions of TMEM165 that have yet to be identified (Krzewinski-Recchi *et al.* 2017).

We also provide evidence that activated calcineurin can inhibit the forward-mode activity of Gdt1 *in vivo*, similar to that of Vcx1: in the presence of the calcineurin inhibitor FK506, *gdt1∆* and *vcx1∆* mutants were far more hypersensitive to Ca^2+^ than in the absence of the calcineurin inhibitor, even when Pmc1 and Crz1 were eliminated. Gdt1 did not mediate the inhibition of Vcx1 by calcineurin, and Vcx1 did not mediate the inhibition of Gdt1 by calcineurin, as calcineurin still retains its inhibitory effects when one or the other transporter has been eliminated. The molecular mechanism(s) by which calcineurin regulates the function of Gdt1 and Vcx1 remain unknown, as neither protein undergoes changes in expression or mobility on SDS-PAGE upon activation/inhibition of calcineurin. Our finding that calcineurin still inhibited reverse-mode activity of Vcx1 in the absence of Vph1 suggests that neither the vacuolar V-ATPase nor luminal H^+^ are key mediators of this regulation. We also ruled out Erd1 as an intermediary of Gdt1 inhibition by calcineurin, as this regulation persisted in *erd1∆ vcx1∆* strains (Table S1). Because Gdt1 and Vcx1 both seem to promote H^+^/Ca^2+^ exchange, it is tempting to speculate that calcineurin regulates the pH of the cytoplasm or organelles in high Ca^2+^ conditions, which would alter the ability of transporters to bind Ca^2+^. Indeed, new evidence suggests that calcineurin may downregulate the plasma membrane H^+^ pump Pma1 (P. Kane, personal communication), potentially causing acidification of the cytoplasm and diminishing forward Ca^2+^ transport by H^+^/Ca^2+^ exchangers.

Because Gdt1 and Vcx1 may also inhibit calcineurin activation by removing Ca^2+^ from the cytoplasm, both proteins have the potential to form double-negative feedback loops with calcineurin (Figure 1A). Double-negative feedback loops generate positive feedback in signaling networks, and tend to promote switch-like transitions between two stable states (Ferrell 2002). In such a scenario, calcineurin is less likely to become activated when Gdt1 and Vcx1 are fully functional, but as cytosolic Ca^2+^ concentrations rise and calcineurin becomes activated, both Gdt1 and Vcx1 may become progressively inhibited, thus accelerating the activation of calcineurin and the further inhibition of H^+^/Ca^2+^ exchangers. Double-negative feedback loops can contribute to heterogeneity in clonal cell populations and a form of cellular memory (hysteresis), as observed previously during the switching between high- and low-affinity Pi transporters in yeast (Wykoff *et al.* 2007). In addition to these advantages, yeast cells may also inhibit the H^+^/Ca^2+^ exchangers in high Ca^2+^ conditions to avoid reverse-mode operation of Vcx1 and Gdt1 and possible futile cycling with the Ca^2+^ ATPases (Pmc1 and Pmr1, which are upregulated by calcineurin signaling). It will be interesting to determine precisely how and why calcineurin inhibits Gdt1 and Vcx1 functions *in vivo*, and whether such regulation contributes to the unexplained “bursts” of free Ca^2+^ elevation and calcineurin signaling that have been observed through real-time imaging in single yeast cells (Cai *et al.* 2008; Carbo *et al.* 2017).

A better understanding of H^+^, Ca^2+^, and Pi homeostasis in the Golgi complex may help explain how this organelle can operate so differently in different tissues of humans. In addition to a general housekeeping function in nonsecretory cells, specialized secretory cells may require huge increases in glycosylation and corresponding increases in byproduct production. In the case of alveolar epithelial cells of the mammary gland, which can produce massive quantities of lactose (a product of glycosylation) and casein micelles (rich in Ca^2+^ and Pi) in the Golgi complex during lactation (Neville 2005), retrieval of the Pi byproduct may not be beneficial and removal of the H^+^ byproduct in exchange for Ca^2+^ may be exceptionally important. Interestingly, to potentially meet this demand, expression of TMEM165 mRNA and protein becomes massively increased in alveolar epithelial cells just as milk production begins (Reinhardt *et al.* 2014). The full repertoire of functions carried out by the Gdt1 and the Erd1 families of proteins will be fascinating to unravel in the many different species and cellular situations.

## Supplementary Material

Supplemental material is available online at www.g3journal.org/lookup/suppl/doi:10.1534/g3.117.300339/-/DC1.
