# Supplementary material for: H+ and Pi Byproducts of Glycosylation Affect Ca2+ Homeostasis and Are Retrieved from the Golgi Complex by Homologs of TMEM165 and XPR1
Source: G3 (Bethesda). 2017 Oct 17;7(12):3913–24. doi: 10.1534/g3.117.300339 (PMC5714488; doi:10.1534/g3.117.300339)
Supplement: Supplementary file 1 [file 3913TableS1.pdf]

Supplemental Table 1. Sensitivity of mutant strains to added CaCl2 (experiment A)

		Strain Names		Genotypes and Conditions [a]							CV [b]	IC50 [c]	Performance Factor [d]						
**EXP**	**LINE**	***MATa***	***MATalpha***	***PMC1***	***VCX1***	***GDT1***	***CRZ1***	***CN***	***CMK2***	***ERD1***	**%**	**mM**	***PMC1***	***VCX1***	***GDT1***	***CRZ1***	***CN***	***CMK2***	***ERD1***
A	1	K1849	K1850	Δ							3	52		0.3	0.2	-0.1	-3.1	-0.3	
A	2	K1851	K1852	Δ			Δ				7	55		0.9	0.3		-3.2	-0.6	
A	3	K1857	K1858	Δ		Δ					0	46		0.6		0.1	-1.0	0.1	
A	4	K1859	K1860	Δ		Δ	Δ				11	44		0.9			-0.9	-0.4	
A	5	K1865	K1866	Δ	Δ						0	43			0.6	0.5	0.3	0.1	
A	6	K1867	K1868	Δ	Δ		Δ				7	30			0.4		0.3	0.4	
A	7	K1873	K1874	Δ	Δ	Δ					12	30				0.4	0.6	-0.2	
A	8	K1875	K1876	Δ	Δ	Δ	Δ				5	23					0.9	0.1	
A	9	K1849	K1850	Δ				+FK			2	464		3.8	2.4	-0.1		0.2	
A	10	K1851	K1852	Δ			Δ	+FK			1	511		4.3	2.6			0.6	
A	11	K1857	K1858	Δ		Δ		+FK			3	90		2.2		0.1		0.1	
A	12	K1859	K1860	Δ		Δ	Δ	+FK			1	84		2.7				0.4	
A	13	K1865	K1866	Δ	Δ			+FK			9	34			0.8	0.4		0.9	
A	14	K1867	K1868	Δ	Δ		Δ	+FK			7	25			1.0			1.3	
A	15	K1873	K1874	Δ	Δ	Δ		+FK			32	19				0.6		0.7	
A	16	K1875	K1876	Δ	Δ	Δ	Δ	+FK			4	13						1.0	
A	17	K1853	K1854	Δ					Δ		4	63		0.6	0.5	-0.4	-2.7		
A	18	K1855	K1856	Δ			Δ		Δ		14	84		1.8	0.5		-2.0		
A	19	K1861	K1862	Δ		Δ			Δ		19	43		0.3		-0.4	-0.9		
A	20	K1863	K1864	Δ		Δ	Δ		Δ		6	57		1.4			-0.1		
A	21	K1869	K1870	Δ	Δ				Δ		2	41			0.2	0.8	1.2		
A	22	K1871	K1872	Δ	Δ		Δ		Δ		1	23			0.1		1.2		
A	23	K1877	K1878	Δ	Δ	Δ			Δ		4	35				0.7	1.6		
A	24	K1879	K1880	Δ	Δ	Δ	Δ		Δ		4	21					1.8		
A	25	K1853	K1854	Δ				+FK	Δ		0	403		4.5	2.3	0.2			
A	26	K1855	K1856	Δ			Δ	+FK	Δ		1	343		5.1	2.5				
A	27	K1861	K1862	Δ		Δ		+FK	Δ		1	82		2.8		0.4			
A	28	K1863	K1864	Δ		Δ	Δ	+FK	Δ		15	62		3.3					
A	29	K1869	K1870	Δ	Δ			+FK	Δ		26	18			0.6	0.8			
A	30	K1871	K1872	Δ	Δ		Δ	+FK	Δ		7	10			0.7				
A	31	K1877	K1878	Δ	Δ	Δ		+FK	Δ		14	12				0.9			
A	32	K1879	K1880	Δ	Δ	Δ	Δ	+FK	Δ		16	6							
A	33	K1833	K1834								16	178	1.8		0.7	1.1	-1.8	-0.1	
A	34	K1835	K1836				Δ				17	81	0.6		0.1		-2.8	-0.5	
A	35	K1841	K1842			Δ					6	107	1.2			0.5	-0.1	0.0	
A	36	K1843	K1844			Δ	Δ				25	75	0.8				-0.4	-0.4	
A	37	K1833	K1834					+FK			6	635	0.5		2.5	0.2		0.3	
A	38	K1835	K1836				Δ	+FK			4	563	0.1		2.5			0.5	
A	39	K1841	K1842			Δ		+FK			9	113	0.3			0.2		0.3	
A	40	K1843	K1844			Δ	Δ	+FK			12	97	0.2					0.4	
A	41	K1837	K1838						Δ		16	195	1.6		0.9	0.8	-1.4		
A	42	K1839	K1840				Δ		Δ		12	112	0.4		0.2		-1.8		
A	43	K1845	K1846			Δ			Δ		29	103	1.3			0.1	0.1		
A	44	K1847	K1848			Δ	Δ		Δ		6	95	0.7				0.4		
A	45	K1837	K1838					+FK	Δ		5	498	0.3		2.4	0.3			
A	46	K1839	K1840				Δ	+FK	Δ		1	403	0.2		2.5				
A	47	K1845	K1846			Δ		+FK	Δ		13	94	0.2			0.4			
A	48	K1847	K1848			Δ	Δ	+FK	Δ		32	73	0.2						

Footnotes

[a] addition of 0.2 μg/mL FK506

[b] coefficient of variation between two strains (in %) or one strain (*)

[c] normalized average of IC50 from two strains (in mM CaCl2)

[d] PF = log base 2 of (IC50 WT strain/IC50 Δ strain)

Supplemental Table 1. Sensitivity of mutant strains to added CaCl2 (experiment B)

		Strain Names		Genotypes and Conditions [a]							CV [b]	IC50 [c]	Performance Factor [d]						
EXP	LINE	*MATa*	*MATalpha*	*PMC1*	*VCX1*	*GDT1*	*CRZ1*	*CN*	*CMK2*	*ERD1*	%	mM	*PMC1*	*VCX1*	*GDT1*	*CRZ1*	*CN*	*CMK2*	*ERD1*
B	1	NS122	NS123	Δ							2	56		0.1	0.0	-0.6	-2.6		-0.1
B	2	NS132	NS133	Δ			Δ				1	84		1.9	0.6		-2.1		-0.1
B	3	NS136	NS137	Δ		Δ					4	57		0.8		0.0	-0.4		-0.1
B	4	NS144	NS145	Δ		Δ	Δ				12	56		1.5			-0.3		0.0
B	5	NS154	NS155	Δ	Δ						0	52			0.6	1.2	1.1		-0.1
B	6	NS164	NS165	Δ	Δ		Δ				4	22			0.1		0.1		-0.7
B	7	NS168	NS169	Δ	Δ	Δ					1	33				0.7	1.3		-0.2
B	8	NS176	NS177	Δ	Δ	Δ	Δ				*	20					0.7		-0.2
B	9	NS122	NS123	Δ				+FK			1	348		3.9	2.2	0.0			0.7
B	10	NS132	NS133	Δ			Δ	+FK			0	347		4.1	2.3				0.7
B	11	NS136	NS137	Δ		Δ		+FK			5	73		2.4		0.0			0.9
B	12	NS144	NS145	Δ		Δ	Δ	+FK			11	71		2.5					0.9
B	13	NS154	NS155	Δ	Δ			+FK			5	23			0.8	0.2			-0.7
B	14	NS164	NS165	Δ	Δ		Δ	+FK			6	20			0.7				-0.4
B	15	NS168	NS169	Δ	Δ	Δ		+FK			0	14				0.2			0.0
B	16	NS176	NS177	Δ	Δ	Δ	Δ	+FK			*	12							0.1
B	17	NS128	NS129	Δ						Δ	1	61		0.1	0.0	-0.5	-1.8		
B	18	NS138	NS139	Δ			Δ			Δ	0	88		1.3	0.7		-1.3		
B	19	NS140	NS141	Δ		Δ				Δ	4	59		0.7		0.1	0.6		
B	20	NS146	NS147	Δ		Δ	Δ			Δ	1	56		1.3			0.5		
B	21	NS160	NS161	Δ	Δ					Δ	1	56			0.6	0.6	0.5		
B	22	NS170	NS171	Δ	Δ		Δ			Δ	0	37			0.7		0.5		
B	23	NS172	NS173	Δ	Δ	Δ				Δ	8	37				0.7	1.4		
B	24	NS178	NS179	Δ	Δ	Δ	Δ			Δ	6	23					1.0		
B	25	NS128	NS129	Δ				+FK		Δ	1	210		2.4	2.5	0.0			
B	26	NS138	NS139	Δ			Δ	+FK		Δ	7	213		3.0	2.5				
B	27	NS140	NS141	Δ		Δ		+FK		Δ	1	38		1.4		0.0			
B	28	NS146	NS147	Δ		Δ	Δ	+FK		Δ	1	38		1.7					
B	29	NS160	NS161	Δ	Δ			+FK		Δ	0	39			1.5	0.6			
B	30	NS170	NS171	Δ	Δ		Δ	+FK		Δ	2	26			1.2				
B	31	NS172	NS173	Δ	Δ	Δ		+FK		Δ	16	14				0.3			
B	32	NS178	NS179	Δ	Δ	Δ	Δ	+FK		Δ	8	12							
B	33	NS116	NS117								3	298	2.4	0.0	0.2	1.4	-0.3		0.1
B	34	NS120	NS121				Δ				0	111	0.4	0.7	0.2		-1.7		0.2
B	35	NS124	NS125			Δ					*	257	2.2	0.4		1.4	1.8		0.6
B	36	NS134	NS135			Δ	Δ				1	94	0.7	0.6			0.0		0.6
B	37	NS148	NS149		Δ						2	291	2.5		0.6	2.1	1.2		0.1
B	38	NS152	NS153		Δ		Δ				8	68	1.6		0.1		-0.8		0.0
B	39	NS156	NS157		Δ	Δ					6	197	2.6			1.7	1.6		0.1
B	40	NS166	NS167		Δ	Δ	Δ				3	62	1.6				0.4		0.1
B	41	NS116	NS117					+FK			0	372	0.1	1.6	2.3	0.0			0.3
B	42	NS120	NS121				Δ	+FK			1	364	0.1	1.6	1.9				0.8
B	43	NS124	NS125			Δ		+FK			*	76	0.1	0.2		-0.3			0.9
B	44	NS134	NS135			Δ	Δ	+FK			1	96	0.4	1.1					1.0
B	45	NS148	NS149		Δ			+FK			1	124	2.4		0.9	0.0			0.1
B	46	NS152	NS153		Δ		Δ	+FK			4	123	2.6		1.4				0.3
B	47	NS156	NS157		Δ	Δ		+FK			2	67	2.3			0.5			1.2
B	48	NS166	NS167		Δ	Δ	Δ	+FK			1	46	1.9						0.9
B	49	NS118	NS119							Δ	2	277	2.2	0.0	0.8	1.5	-0.1		
B	50	NS126	NS127				Δ			Δ	2	97	0.1	0.5	0.6		-1.2		
B	51	NS128	NS129			Δ				Δ	2	164	1.5	-0.1		1.4	2.0		
B	52	NS142	NS143			Δ	Δ			Δ	2	64	0.2	0.1			0.4		
B	53	NS150	NS151		Δ					Δ	3	272	2.3		0.6	2.0	1.2		
B	54	NS158	NS159		Δ		Δ			Δ	3	67	0.9		0.2		-0.6		
B	55	NS162	NS163		Δ	Δ				Δ	*	178	2.3			1.6	2.6		
B	56	NS174	NS175		Δ	Δ	Δ			Δ	4	59	1.3				1.3		
B	57	NS118	NS119					+FK		Δ	2	297	0.5	1.4	2.9	0.5			
B	58	NS126	NS127				Δ	+FK		Δ	4	215	0.0	1.1	2.2				
B	59	NS128	NS129			Δ		+FK		Δ	3	41	0.1	0.5		-0.2			
B	60	NS142	NS143			Δ	Δ	+FK		Δ	29	48	0.3	1.0					
B	61	NS150	NS151		Δ			+FK		Δ	1	115	1.6		2.0	0.2			
B	62	NS158	NS159		Δ		Δ	+FK		Δ	3	103	2.0		2.1				
B	63	NS162	NS163		Δ	Δ		+FK		Δ	*	29	1.0			0.2			
B	64	NS174	NS175		Δ	Δ	Δ	+FK		Δ	9	25	1.1						

Footnotes

[a] addition of 0.2 µg/mL FK506

[b] coefficient of variation between two strains (in %) or one strain (*)

[c] normalized average of IC50 from two strains (in mM CaCl2)

[d] PF = log base 2 of (IC50 WT strain/IC50 Δ strain)
