# Supplementary material for: H+ and Pi Byproducts of Glycosylation Affect Ca2+ Homeostasis and Are Retrieved from the Golgi Complex by Homologs of TMEM165 and XPR1
Source: G3 (Bethesda). 2017 Oct 17;7(12):3913–24. doi: 10.1534/g3.117.300339 (PMC5714488; doi:10.1534/g3.117.300339)
Supplement: Supplementary file 2 [file 3913TableS2.pdf]

Supplemental Table 2. List of yeast strains used in this study

Strain	Background	Genotype	Source
K601	W303 [a]	*MATa*	Cunningham and Fink (1996)
K605	W303	*MATa pmc1::TRP1*	Cunningham and Fink (1996)
K661	W303	*MATa vcx1Δ*	Cunningham and Fink (1996)
K665	W303	*MATa pmc1::TRP1 vcx1Δ*	Cunningham and Fink (1996)
K1801	W303	*MATa*	This Study
K1802	W303	*MATa stv1::KanR*	This Study
K1803	W303	*MATa vph1::NatR*	This Study
K1804	W303	*MATa vph1::NatR stv1::KanR*	This Study
K1805	W303	*MATa gdt1::HIS3*	This Study
K1806	W303	*MATa gdt1::HIS3 stv1::KanR*	This Study
K1807	W303	*MATa gdt1::HIS3 vph1::NatR*	This Study
K1808	W303	*MATa gdt1::HIS3 vph1::NatR stv1::KanR*	This Study
K1809	W303	*MATa vcx1Δ*	This Study
K1810	W303	*MATa vcx1Δ stv1::KanR*	This Study
K1811	W303	*MATa vcx1Δ vph1::NatR*	This Study
K1812	W303	*MATa vcx1Δ vph1::NatR stv1::KanR*	This Study
K1813	W303	*MATa vcx1Δ gdt1::HIS3*	This Study
K1814	W303	*MATa vcx1Δ gdt1::HIS3 stv1::KanR*	This Study
K1815	W303	*MATa vcx1Δ gdt1::HIS3 vph1::NatR*	This Study
K1816	W303	*MATa vcx1Δ gdt1::HIS3 vph1::NatR stv1::KanR*	This Study
K1817	W303	*MATα*	This Study
K1818	W303	*MATα stv1::KanR*	This Study
K1819	W303	*MATα vph1::NatR*	This Study
K1820	W304	*MATα vph1::NatR stv1::KanR*	This Study
K1821	W303	*MATα gdt1::HIS3*	This Study
K1822	W303	*MATα gdt1::HIS3 stv1::KanR*	This Study
K1823	W303	*MATα gdt1::HIS3 vph1::NatR*	This Study
K1824	W303	*MATα gdt1::HIS3 vph1::NatR stv1::KanR*	This Study
K1825	W303	*MATα vcx1Δ*	This Study
K1826	W303	*MATα vcx1Δ stv1::KanR*	This Study
K1827	W303	*MATα vcx1Δ vph1::NatR*	This Study
K1828	W303	*MATα vcx1Δ vph1::NatR stv1::KanR*	This Study
K1829	W303	*MATα vcx1Δ gdt1::HIS3*	This Study
K1830	W303	*MATα vcx1Δ gdt1::HIS3 stv1::KanR*	This Study
K1831	W303	*MATα vcx1Δ gdt1::HIS3 vph1::NatR*	This Study
K1832	W303	*MATα vcx1Δ gdt1::HIS3 vph1::NatR stv1::KanR*	This Study
K1833	W303	*MATa*	This Study
K1834	W303	*MATα*	This Study
K1841	W303	*MATa gdt1::HIS3*	This Study
K1842	W303	*MATα gdt1::HIS3*	This Study
K1849	W303	*MATa pmc1::LEU2*	This Study
K1850	W303	*MATα pmc1::LEU2*	This Study
K1857	W303	*MATa pmc1::LEU2 gdt1::HIS3*	This Study
K1858	W303	*MATα pmc1::LEU2 gdt1::HIS3*	This Study
K1865	W303	*MATa vcx1Δ pmc1::LEU2*	This Study
K1866	W303	*MATα vcx1Δ pmc1::LEU2*	This Study
K1873	W303	*MATa vcx1Δ pmc1::LEU2 gdt1::HIS3*	This Study
K1874	W303	*MATα vcx1Δ pmc1::LEU2 gdt1::HIS3*	This Study
K1883	W303	*MATα vcx1Δ pmc1::TRP1 crz1::KanR*	This Study
CTS05	W303	*MATa*	This Study
CTS06	W303	*MATα*	This Study
CTS09	W303	*MATa gdt1::HIS3*	This Study
CTS10	W303	*MATα gdt1::HIS3*	This Study
CTS13	W303	*MATa pmr1::LEU2*	This Study
CTS14	W303	*MATα pmr1::LEU2*	This Study
CTS17	W303	*MATa pmr1::LEU2 gdt1::HIS3*	This Study
CTS18	W303	*MATα pmr1::LEU2 gdt1::HIS3*	This Study
AK011	W303	*MATa vph1::NatR*	This Study
AK012	W303	*MATa pmc1::TRP1 vph1::NatR*	This Study
AK013	W303	*MATa pmc1::TRP1 vcx1Δ* vph1::NatR	This Study
AK014	W303	*MATa vcx1Δ vph1::NatR*	This Study
NS001	W303	*MATa vcx1Δ*	This Study
NS002	W303	*MATα vcx1Δ*	This Study
NS118	W303	*MATa erd1::NatR*	This Study
NS119	W303	*MATα erd1::NatR*	This Study
NS185	W303	*MATa pho84::TRP1*	This Study
NS186	W303	*MATα pho84::TRP1*	This Study
NS187	W303	*MATa erd1::NatR pho84::TRP1*	This Study
NS188	W303	*MATα erd1::NatR pho84::TRP1*	This Study
EY916	W303	*MATa pho84Δ::HIS3 pho87Δ::CgHIS3 pho89Δ::CgHIS3 pho90Δ::CgHIS3 pho91Δ::KlURA3*	Wykoff and O'Shea (2001)
NS229	W303	*MATa pho84Δ::HIS3 pho87Δ::CgHIS3 pho89Δ::CgHIS3 pho90Δ::CgHIS3 pho91Δ::KlURA3 erd1::NatR*	This Study
TSA36	BY4741	MATa sec1-1::KanR	Li et al. (2011)
NS227	BY4741	*MATa sec1-1::KanR erd1::NatR*	This Study
	BY4741	*MATa VRG4-TAP-AID-6xFLAG::TIR1::URA3*	This Study
NS228	BY4741	*MATa VRG4-TAP-AID-6xFLAG::TIR1::URA3 erd1::NatR*	This Study

[a] background mutations in W303 (*leu2-3,112 trp1-1 can1-100 ura3-1 ade2-1 his3-11,15)*

[b] background mutations in BY4741 (his3Δ1 leu2Δ0 met15Δ0 ura3Δ0)
